# Role of Functional Beverages on Sport Performance and Recovery

**DOI:** 10.3390/nu10101470

**Published:** 2018-10-10

**Authors:** Stefania Orrù, Esther Imperlini, Ersilia Nigro, Andreina Alfieri, Armando Cevenini, Rita Polito, Aurora Daniele, Pasqualina Buono, Annamaria Mancini

**Affiliations:** 1 Dipartimento di Scienze Motorie e del Benessere, Università degli Studi di Napoli “Parthenope”, via Medina 40, 80133 Napoli, Italy; orru@uniparthenope.it (S.O.); andreina.alfieri@uniparthenope.it (A.A.); buono@uniparthenope.it (P.B.); 2 IRCCS SDN, via E. Gianturco 113, 80142 Napoli, Italy; esther.imperlini@unina.it; 3 Ceinge-Biotecnologie Avanzate S.c.a r.l., Via G. Salvatore 486, 80145 Napoli, Italy; nigro@ceinge.unina.it (E.N.); armando.cevenini@unina.it (A.C.); rita.polito@unicampania.it (R.P.); aurora.daniele@unicampania.it (A.D.); 4 Dipartimento di Medicina e di Scienze della Salute “Vincenzo Tiberio”, Università degli Studi del Molise, 86100 Campobasso, Italy; 5 Dipartimento di Medicina molecolare e Biotecnologie mediche, Università degli Studi di Napoli “Federico II”, via S. Pansini 5, 80131 Napoli, Italy; 6 Dipartimento di Scienze e Tecnologie Ambientali Biologiche Farmaceutiche, Università della Campania “Luigi Vanvitelli”, Via G. Vivaldi 42, 81100 Caserta, Italy

**Keywords:** functional beverages, sports drinks, hydration, dehydration, CHO-enriched beverages, lipid-enriched beverages, protein-enriched beverages

## Abstract

Functional beverages represent a palatable and efficient way to hydrate and reintegrate electrolytes, carbohydrates, and other nutrients employed and/or lost during physical training and/or competitions. Bodily hydration during sporting activity is one of the best indicators of health in athletes and can be a limiting factor for sport performance. Indeed, dehydration strongly decreases athletic performance until it is a risk to health. As for other nutrients, each of them is reported to support athletes’ needs both during the physical activity and/or in the post-workout. In this study, we review the current knowledge of macronutrient-enriched functional beverages in sport taking into account the athletes’ health, sports performance, and recovery.

## 1. Introduction

Functional foods are defined by the European Commission as “a food product that can only be considered functional if together with the basic nutritional impact it has beneficial effects on one or more function of the human organism thus either improving the general physical conditions or/and decreasing the risk of the evolution of disease” [1]. Among the different types of functional foods freely available on the market, beverages are the most popular; in fact, they easily meet consumers’ demands in terms size, shape, storage, and possibility to contain desirable nutrients and bioactive compounds [2,3,4]. Over the past decades, the functional beverage market has developed an increasing number of products, estimated in $US billions and advertised for distinguishing features, such as to improve gut or cardiovascular health, to support the immune system, to help in weight management, or to counteract aging processes [5]. In this scenario, there are drinks containing omega-3 fatty acids and CoQ10 for heart health, fiber and probiotics for both gut health and weight management, collagen for improving overall skin appearance and vitamin D, and zinc for enhanced immunity. Functional ingredients also include also flavors, sweeteners, stabilizers, and colors [5,6,7].

Intense marketing efforts are continually made to encourage the consumption of functional beverages, even when they are not needed [8]. A crucial issue is represented by food labels, which by means of health and nutrient claims, should protect consumers from misleading information. Many functional products have clean safety histories, but sometimes labels might not contain the right amount of the listed items, or miss some extra-ingredients, or be accidentally contaminated with allergens, and more concerns arise when youngers or intolerant subjects are targeted. Moreover, sometimes claimed substances have no scientifically proven effects, or their concentration is below the optimal concentration. The Food and Drug Administration (FDA) in the US and the European Food Safety Authority (EFSA) in Europe regulate which health claims are approved and may be put on labels [9]. Although their aims are similar, their regulations are not exactly overlapping. A main difference is related to the FDA ban prohibiting claims about the diagnosis, cure, mitigation, or treatment of disease on food labels; conversely, European regulation does not include such a ban but authorizes health claims based on scientific evidence and is easily understood by consumers [9]. FDA recognizes four different types of healthy claims: Nutrient content, authorized health, qualified health, and structure/function claims; however, in the US functional food has not got a statutory definition and it is generalized as a food or supplement. So, only manufacturers are responsible for the safety of their products, for the all the ingredients listed on the label, and for claims without empirical evidence, as long as there is a disclaimer. On the other hand, EFSA allows two categories of claims: Nutrition and health claims. The former provides information on the energy value and on the amount of nutrients contained (or not contained); the latter are subdivided into three types: Function health claims, risk reduction claims, and claims referring to children’s development. Unlike the FDA, the EFSA has the responsibility to guarantee consumers’ safety by prohibiting any false advertising; hence, although a disclaimer is not put on the label, the EFSA ensures that every claim passes a threshold of criteria [9]. In general, functional beverages can be distinguished as (i) dairy-based beverages, including probiotics and mineral-enriched drinks, (ii) vegetable and fruit beverages and (iii) sports and energy drinks [10]. In this study, we deal with sports drinks, that usually do not include caffeine or other stimulants, unlike energy drinks, and that account for about 30% of market share breakdown of the functional beverage category in the US [8,11].

Functional sports drinks play an important role in hydrating, in improving athletic performance, and in preventing or helping specific health conditions [12]. Their formulas can be designed specifically to increase energy, to improve mental focus and/or to prevent bone and joint pain [6]. In the sports context, the principal function of such drinks is to hydrate athletes and restore the electrolytes, carbohydrates (CHO), and other nutrients which can be depleted during exercise [7]. Sports drinks are developed using essential electrolytes like sodium, potassium, chloride, calcium, phosphate, and magnesium, which are lost by sweating during training and/or competition [7]. Amino acids are used to slow fatigue and improve muscle function, whilst B vitamins are used to boost metabolism and generate energy. Simple CHO can be used for a quick energy burst, whereas complex CHO provide sustained energy.

In the present review, we will analyze the current knowledge on hydration and dehydration in relation to physical exercise and we will report data about functional beverages in sport considering both athletes’ health and sports performance.

## 2. Hydration in Sport

Water can be considered as an essential nutrient in diet. The importance of this component in the human daily diet is given by the fact that our body is mainly made up of water (about 70% in an adult and 80% in children) [5,13,14]. Therefore, it is crucial for health to keep the total body water (TBW) content within the proper values. It is still a controversy on the right amount of water to assume, considering the several factors that influence it. The signal for water intake is mainly thirst. The thirst sensation is regulated by the central nervous system, which receives signals relevant to hydration status from both central and peripheral pathways. In fact, dehydration corresponds to changes in the osmolality, as well as in the volume of the blood system. The first ones are sensed by the *organum vasculosum* of the lamina terminalis (OVLT), which sends signals to the hypothalamus that in turn, stimulates thirst and vasopressin release. Changes in blood volume are detected by atrial baroreceptors which induce drinking through the median preoptic nucleus (NM) [12].

Similar to calories, the right amount of water to be drunk is dictated by the balance between the intake and the losses. The intake comes from both fluids and solid foods [7]. Fruits and vegetables are the major source of water from food. Water coming from oxidation of macronutrients also makes a contribution, although often negligible. Water loss from the body occurs through the urinary system, the skin, the gastrointestinal tract, and the respiratory surfaces [15].

Bioelectrical impedance analysis (BIA) is the gold standard to evaluate body hydration. By measuring the resistance to a low electrical current as it goes through the body, BIA is able to distinguish between the two major body compartments, the body fat mass (FM) and the fat-free mass (FFM), where the former is a non-conducting tissue unlike the latter. BIA also allows the differentiation between intracellular and extracellular water compartments within TBW [16,17].

The World Health Organization (WHO) defines physical activity as any bodily movement produced by skeletal muscles that requires energy expenditure [18]. Physical activity improves health, since it has been associated with reduced risk of coronary heart disease, obesity, type 2 diabetes, and other diseases. On the other side, physical inactivity has been identified as the fourth leading risk factor for global mortality (6% of deaths globally) [18,19,20]. The guidelines about physical activity have changed during the last three decades as they consider changes in lifestyle. There are several differences between professional and amateur athletes, where the first ones need to maintain a very high standard of training. It follows that professional athletes undergo much higher and harder workouts, depending on many factors such as the type of sport training, the number and the duration of sessions, the specific environment setting, etc. [18,19]. Both amateur and professional athletes may experience dehydration; however, there is confusion about the need of integrating sport drinks into the diet or just paying attention to the amount of consumed water [5,12,20]. What experts do agree on is that for most people just drinking water is sufficient to rehydrate [6,12].

The effect of body water balance on exercise performance has been extensively researched. Water balance can influence not only endurance performance but also power and strength [7,12,21]. Athletes should drink before they become thirsty to maximize endurance performance, in fact, it is crucial to be hydrated before exercise. A well-hydrated state ensures a normal plasma osmolality level, hence minimizing thirst at the start of exercise.

As for the real drinking need during exercise sessions, data are scattered. Holland and colleagues reported that fluid consumption is linked to the duration of the physical activity for cyclists. During high-intensity cycling exercise (about 80% VO_2_max, <1 h) fluid consumption is associated with reductions in the performance; at moderate intensity (60–70% VO_2_max, ranging from 1 to 2 h), cyclists should expect a gain in performance of at least 2% if fluid is integrated; for cycling exercise longer than 2 h, conducted at moderate intensity, fluid improves performance by at least 3% [22]. Bergeron et al. suggested good rehydration to tennis players after a match or in between two matches [23]. For football players, fluid intake is recommended before the match and during the breaks to avoid dehydration [24]. The volume of drink consumed is critical for ensuring a return to euhydration [24].

Through sweating, athletes lose water and electrolytes, in particular sodium and chloride and a smaller amount of potassium. During physical activity, electrolytes are fundamental as they perform different biological functions, in particular sodium and potassium regulate the amount of body water, the former being involved in muscle excitability and cellular permeability, the latter in protein and CHO synthesis. Chloride, on the other hand, maintains osmotic pressure and acid-base balance and is an essential component of gastric juice [12,25]. Compared to other body fluids, sweat is hypotonic and its composition is influenced by many factors, such as the rate of sweating, diet, and acclimatization, all of them depending from inter-individual variability [12]. Since there is a potential link between salt loss and muscle cramps, it is important to identify those athletes prone to muscle cramps due to the loss of large amounts of salts during exercise [26]. In contrast, high salt intake in the diet can negatively affect blood pressure and cardiovascular risk, so not all athletes should consume a high sodium diet or drink during exercise. According to the American College of Sports Medicine (ACSM), for physical activity shorter than 3h an isotonic drink (0.5–0.7 g/L Na^+^) should be assumed, whilst for physical activity longer than 3h a more concentrated drink is recommended (0.7–1 g/L Na^+^) [27].

Dehydration during exercise induces weight loss that can range between 1 and 3% [25,28]. There are no clear and unique data about how dehydration and the consequent weight loss may affect the performance [23]. However, most studies suggest that if dehydration and weight loss are kept under 2% during training sessions, they do not affect the performance of well hydrated athletes who reintegrate liquid loss afterwards [7,12,13,14,15,21]. Dehydration to the extent of 2–7% of body mass negatively affects endurance exercise performance in cycling time-trial type exercise; in marathon and triathlon races, the effects of dehydration could change [16]. In this regard, a significant linear relationship between the degree of body weight loss and race finish time has been demonstrated, with the greatest body weight loss being positively related to the best racing times [12,22,29,30].

The main factor influencing the performance of athletes in response to hydration is the environment temperature. In temperate climate, dehydration by 1–2% of body mass has no effect on endurance performance when the exercise duration is around 90 min, but performance is impaired when the level of dehydration is higher than 2% of body mass and the exercise duration is longer than 90 min [14]. In a hotter environment (31–32 °C), dehydration equivalent to 2% body mass loss during exercise impairs endurance performance, whereas in cold environments a body mass loss >2% may be tolerable for endurance exercise [20].

Sometimes an athletes’ goal is to lose weight through dehydration for an advantage in sports and competition [30,31]. Unsafe weight loss methods through aggressive nutritional strategies are an issue for competitive athletes and active adults. Such an aim can be achieved by means of different protocols: Active dehydration, induced by an excessive sweating during exercise while wearing heavy clothing, and passive dehydration through food restriction and diuretics promoting fluid loss [30,31,32,33].

Field expedient measures to assess hydration status are available, such as (i) body mass; (ii) blood biomarkers; (iii) urine-specific gravity and color, and (iv) sensation of thirst [15,16,34,35].

(i) When using changes in body mass, it is assumed that the acute loss of 1g is equivalent to the loss of 1mL of water. This method is most effective when the pre-exercise baseline body mass is measured in a well hydrated individual. Generally, this method is used to evaluate hydration status at the end of exercise sessions [15,16,34].

ii. During exercise, athletes often experience water deficit through sweating (involuntary dehydration). Given sweat is hypotonic, exercise-induced dehydration leads mainly to a hypertonic hypovolemia due to water loss from the plasma. Blood biomarkers of hemoconcentration, such as blood osmolality and sodium levels, have thus been widely used as an index of dehydration. However, between the two, blood osmolality is the most sensitive marker in that it can change even for small variations in hydration status. Generally, blood biomarkers are used to evaluate hydration status of athletes before competitions because the intake of great amounts of fluids during/post exercise sessions/matches can affect these markers [15,16,34].

iii. Urine-specific gravity and color is easily measured in a field setting. However, these parameters can be easily confounded when proper controls are not employed, such as when they are obtained during periods of rehydration or after exercise when glomerular filtration rate has been reduced. However, use of the first morning void following an overnight fast minimizes confounding factors and maximizes measurement reliability. Generally, this method is used to evaluate hydration status before exercise sessions [15,16,34].

iv. Sensation of thirst is a qualitative tool that can be used for hydration assessment. Generally, this method is used to evaluate hydration status during exercise sessions.

Among the above quoted methods, blood or urine biomarkers can be more precise and accurate [15,16,34]. Nevertheless, recently, more samples have been proposed to evaluate the hydration status, such as saliva osmolality, saliva flow rate, sweat and tears. These methods could represent useful improvements in the measurement of hydration status in sport, since sample collection is less invasive; on the other hand, the sensitivity has yet to be determined [15,34].

## 3. Functional Beverages Containing CHO

Functional sports drinks have the ability to stimulate energy processes and compensate for the loss of nutrients and fluids during physical activity; in the last two decades they have become increasingly popular. CHO are the main components of functional sports drinks, both for the best energy yield per mole of oxygen compared to proteins and fats and because they improve physical performance by delaying the depletion of muscle glycogen [36,37]. Several studies have shown that a little amount of CHO (20 g/h) is sufficient to obtain a benefit in sports performance. In fact, Fielding and colleagues observed improvements in performance when 22 g of CHO were ingested every hour, whilst no effect was observed when 11 g/h were consumed [38]. Moreover, Maughan et al. showed that the intake of 16 g/h of glucose (Glc) improves the resistance by 14% [16]. Successively, it was demonstrated that the oxidation rate of exogenous CHO can never be higher than 60 g/h [39], thus determining the upper limit for CHO uptake during exercise. Nowadays, the latest guidelines from the American College of Sports Medicine (ACSM) indicate that during exercise a CHO intake of 30–60 g/h is suggested [40].

CHO are an important energy substrate and their use is determined by the intensity and duration of exercise [5,41], along with training and nutritional status [42,43]. In fact, it has been shown that CHO intake produces an anti-fatigue effect by maintaining high levels of Glc in the blood supporting muscle energy production during physical activity, and if muscle glycogen is running low. Therefore, the integration of CHO through the use of sport drinks is of great importance to maintain optimal sports performance [36]. Large amounts of research in this field has investigated the role of CHO in sports endurance. Temesi and colleagues systematically reviewed 50 randomized controlled trials, evaluating the effects of CHO ingestion during endurance training lasting >1 h [44]. These studies were classified according to 4 types of performance measures: Time to exhaustion (TTE), time trial (TT), submaximal exercise followed by TTE (submax + TTE), and submaximal exercise followed by TT (submax + TT). In all of the different protocols, the ingestion of CHO at 30–80 g/h (6–8% CHO-electrolyte drinks) improved performance parameters by about 2.0% in TTE, 15.1% in TT, 7.5% in submax + TTE, and 54.2% in submax + TT, compared to a placebo. Similar and consistent results were achieved even when the participants were fasting for 8 h or when they took a quick packaged meal; furthermore, the beneficial effects of CHO ingestion were evident when food intake was not controlled in the previous 24 h, thus strengthening its ergogenic effect during endurance exercise [44].

CHO ingestion during resistance training (RT) produced more contrasting results. It is well known that both during and after RT sessions a significant decrease in the muscle glycogen from the lower limbs is observed [45,46,47], thus giving muscle glycogen a key role as an energy substrate, especially during high intensity workouts. More recently, Haff et al. [48] demonstrated an improvement in performance in a second training session (squat performance to exhaustion), held on the same day, with CHO supplement before, during, and after RT. Similar increases in performance for the lower body were reported when CHO supplementation occurred before and during RT. [49,50]. Nevertheless, other studies showed no change in muscle performance when CHO were assumed before and during acute and high-intensity RT [51,52]. Such conflicting results can be correlated to the type of protocol used, to the targeted muscle groups, and to the duration of the RT. Moreover, in addition to strength and conditioning, other aspects of RT, such as jumps and sprints, were also investigated. As for the former, some authors demonstrated an improvement in vertical jump performance when participants used CHO-enriched integrators [53,54]; moreover, this result was not confirmed on vertical leap by others [55,56,57]. Additionally, in sprint performance the supplementation of a CHO drink (6%) provided a significant degree in sprint time during specific soccer training games, [58,59], or no improvement in sprint performance during basketball activities [55,56]. These contradictory data strongly support the need for more robust studies to establish the effectiveness of CHO integration during RT.

During fasting, CHO sources, able to sustain muscle workout, are muscle glycogen and plasma Glc, the latter essentially deriving from hepatic glycogen [60,61]. The ability to store CHO in humans is limited, in fact, less than 3000 kcal of CHO versus 100,000 kcal for lipids are stored in a 75 kg man with 15% body fat [62], so that glycogen stores can be almost completely exhausted within 45–90 min of moderate to intense exercise [63,64], resulting in fatigue strongly associated with the depletion of reserves of endogenous CHO [65,66]. Nutritional strategies to supplement or replace endogenous CHO stores as fuel during exercise have been studied for decades [67]. It is now established that ingestion of CHO during exercise improves endurance performance and delays fatigue in moderate or high intensity exercises performed longer than 45 min [68]. The main factor determining the recovery time is the glycogen resynthesis rate, which is particularly important when the repletion periods are short, such as during periods of intensive training, stage races, and tournament-style competitions. In the post exercise, CHO ingestion is crucial to restore muscle glycogen stocks [69] and the proper ingested amount (g/h) can speed up reintegration [70,71].

Dietary CHO include monosaccharides such as Glc, fructose (Fru), and galactose; disaccharides such as maltose, sucrose (Suc), and lactose; and polysaccharides such as maltodextrin (MD) and starch. Several studies have shown that the type of ingested CHO does not appear to be critical for Glc homeostasis during prolonged exercise and for improving endurance capacity [72], although the assumption of CHO other than Glc monomer can be convenient. In particular, replacing Glc monomer with Glc polymers could allow a rise in CHO content without increasing osmolality [36].

As for the osmotic pressure, sports drinks can be classified into 3 types: Hypotonic, isotonic, and hypertonic. The main determinants influencing the osmotic pressure of CHO-based beverages are the concentration and the molecular weight of CHO. In fact, CHO molecular weight influences gastric emptying and the rate of muscle glycogen replenishment. An optimal CHO-containing sports drink should induce low osmotic pressure with good intestinal absorption. In this regard, oligosaccharides are better than mono- and di-saccharides because they can increase the CHO content, while keeping the drink at a relatively low osmotic pressure [36].

The rate of CHO digestion, intestinal absorption, and hepatic metabolism dictate CHO availability for the muscle tissue, influencing the nutritional choices when the goal is to maximize CHO supply during and after exercise [61]. The hydrolysis of most polysaccharides and disaccharides is rapid and neither digestion nor absorption are limited. Therefore, the Glc polymers (maltose, MD, starch) are digested, absorbed, and used at a similar rate to Glc monomeric [73,74,75]. If the Suc is considered, its intestinal absorption rate is faster than its monomeric components; however, this is not true for its isomer isomaltulose. As a matter of fact, isomaltulose is hydrolyzed slower than Suc due to the different chemical link between Glc and Fru [76]. In particular, isomaltulose produces a lower glycemic and insulinemic response after ingestion, with a lower effect on the inhibition of fat oxidation compared to Suc [77]. On the other hand, isomaltulose exasperates the gastrointestinal disturbance when consumed in large quantities during exercise [78].

In many studies it has been shown that during any type of exercise, in which Glc is ingested alone, the oxidation rate of exogenous CHO is positively related to CHO ingestion rate in a curvilinear fashion, reaching a peak of about 1.2 g/min [79,80,81,82,83,84,85,86,87,88,89]. When Fru is added to Glc or ingested as Suc, oxidation rates around 1.7 g/min can be obtained [61,74,82,87,90]. This could be due to the fact that when large amounts of Glc polymers are ingested, intestinal Glc transporter (SGLT1) can be saturated (1.2 g/min), limiting intestinal absorption [79]; however, free- or Suc-released Fru uses a different intestinal transporter (GLUT-5) and does not compete in the saturation of SGLT1. Furthermore, a disaccharidase-dependent transport mechanism is responsible for Suc absorption and allows the direct transfer of its monomeric units through the brush edge membrane [73].

The main advantage for athletes to drink a Glc-Fru mixture, during an exercise, is the capacity to absorb a greater amount of exogenous CHO in the systemic circulation, which can be used immediately as energy fuel or can be directed toward the liver or the muscle glycogen stocks. Furthermore, isotonic Glc-Fru mixtures cause less intestinal problems than Glc alone, probably due to their faster digestion and absorption, explaining some of their beneficial effects on sports performance [91].

On the other hand, considering the post-exercise regeneration of muscle glycogen, Wallis et al. showed that the ingestion of Glc or Glc/Fru (2/1 ratio), at a rate of 90 g/h, brought similar rates of muscle glycogen synthesis during 4 h post-exercise recovery [92]. Likewise, Trommellen and colleagues also showed that the combined ingestion of Glc and Fru (1.2 g/kg/h Glc plus 0.3 g/kg/h Fru or 0.9 g/kg/h Glc +0.6 g/kg/h Suc) did not further accelerate post-exercise synthesis of muscle glycogen, compared to ingestion of 1.5 g/kg/h of Glc alone [91]. Hence, these data clarify that the post exercise muscle glycogen regeneration rate is independent from the type and the length of ingested CHO; but the combined ingestion of Glc and Fru or Suc results in less gastrointestinal disturbance compared to Glc alone, due to a lower accumulation of CHO in the gastrointestinal tract, in line with previous studies [91,93].

The ingestion of Glc-Fru mixtures or Suc, even if it does not accelerate the resynthesis of muscle glycogen, is able to improve the regeneration of liver glycogen, which is essential to minimize exercise-induced hypoglycemia [94] and ameliorates exercise capacity [64]. Indeed, in previous works it has been proven that ingestion of Fru after exercise favors liver glycogen resynthesis at higher rates compared to Glc ingestion [91,94]. Such a feature must be kept in mind when the athlete competes twice a day in almost consecutive races/matches [64]. Unfortunately, data on this topic are limited due to the invasiveness of the protocols able to determinate liver glycogen content.

The limit of these type of studies is related to the sports drinks which are often obtained in the authors’ laboratories; hence, the results are not always applicable to commercial functional beverages. As a consequence, there is still the need to validate marketing claims on the real beneficial effects of commercially available sport drinks on sports performance. In this regard, Roberts and colleagues investigated the potential influence of commercially available MD/Fru and MD alone (High 5 Ltd, Brighton, UK) at a relatively high CHO concentration (102 g/h) for a race longer than 2 h versus placebo drinks [87]. The authors reported higher increases in oxidation rates of total and exogenous CHO with MD/Fru beverages compared to isoenergetic MD and placebo drinks. In addition, they also reported a significant increase in total fluid delivery, as assessed via plasma ^2^H_2_0 enrichment, with the use of the MD/Fru formula compared to MD and placebo. However, the results from the MD drink were always significantly lower than those from the placebo. The most likely explanation is that the ingestion of MD/Fru resulted in an overall increase of total and exogenous CHO, particularly in the last 30 min of the exercise. Since saturation of the SGLT1 transporter can occur with MD, fluid absorption through the intestinal lumen may be limited. However, the inclusion of Fru reduced SGLT1 saturation and permitted the continued absorption of fluids. These results agreed with the study by Jentjens and collegues, where a Glc-Fru drink determined a higher availability of liquids compared to a beverage containing only an isoenergetic amount of Glc, during a physical exercise carried out at high temperatures [83]. Likewise, Jeukendrup and colleagues compared the effects of water, 8.6% Glc solution, and 8.6% Glc/Fru solution (2/1 ratio) ingestion on gastric emptying and fluid delivery during exercise at moderate intensity; the authors showed that the Glc/Fru solution increased gastric emptying and “fluid delivery” compared to a Glc solution [95].

Therefore, according to these considerations, for prolonged physical activity (>2 h) athletes should prefer drinks with the combination of different CHO to maximize the intake of CHO and fluids, both elements supporting improved exercise performance.

## 4. Functional Beverages Containing Lipids

Among sports drinks, in the last decade, there has been a great increase in the development of functional lipid-enriched beverages containing healthier oils. Such a development was consequent to a significant shift in the consumption of relatively unhealthy fats, rich in saturated and trans fats, in favor of healthy oils, rich in unsaturated fatty acids, but chemically more unstable, because they are prone to oxidation [10,96]. In particular, the dietary supplementation of such functional lipid-enriched beverages has become a very common habit in sports nutrition [1,96,97]. As a matter of fact, a wide range of functional lipid-enriched drinks are commercially available for sportsmen to improve their health and athletic performance, including drinks containing ω-3 polyunsaturated fatty acids (n-3 PUFAs), such as docosahexaenoic acid (DHA), α-linolenic acid (ALA), eicosapentaenoic acid (EPA), and docosapentaenoic acid (DPA) [98,99,100,101,102].

n-3 PUFAs are abundant in plant oils and in marine sources such as salmon, shellfish, and fish oil. They are called “essential” fatty acids, because they cannot be easily synthesized by the organism [102]. The beneficial effects of n-3 PUFAs on health are mainly related to their immunomodulatory and anti-inflammatory properties and their influence on immune function. These properties could explain the positive effects of this fat supplementation in the prevention of many inflammatory diseases, cancer, diabetes, and cardiovascular disease [103,104,105,106,107].

These n-3 PUFA-enriched sports drinks also contain antioxidants, such as vitamin E and polyphenols, to prevent lipid oxidation [99]. As an example, some functional lipid-based beverages contain nut or almond oils, rich in essential nutrients, such as calcium and potassium, and in particular niacin, alpha-tocopherol, and polyphenols [108,109,110].

Scientific data published so far highlights the beneficial effects of dietary supplementation with n-3 PUFAs on athletic performance [111] and on oxidative balance [101]. n-3 PUFAs could be considered important molecules able to modulate both the immune and inflammatory systems, as well as to adapt the response to oxidative stress induced by physical exercise [111,112]. In addition, other studies have demonstrated the beneficial effects of n-3 PUFAs-enriched beverage supplementation during physical exercise on some physiological parameters, such as the deformability of red blood cells (RBC) and their facilitated transport through a greater dilatation of the brachial artery, with consequent increase in blood flow [97,113,114].

However, despite scientific evidence, the effectiveness of such functional lipid-enriched drinks to improve athletic performance is not completely clarified. Moreover, the effects of n-3 PUFAs on the immune response associated with physical activity are still to be elucidated [110,115].

The anti-inflammatory effects of n-3 PUFAs-enriched beverages seem to be mediated by the nuclear factor κβ (NFκβ) signaling pathway, which has a key role in inflammatory response [116]. The expression of pro-inflammatory genes modulated by NFκβ decreases after twelve weeks of n-3 PUFAs-enriched beverage supplementation [117,118]. Another putative mechanism able to explain the anti-inflammatory effects of n-3 PUFAs seems to be related to the acid arachidonic (AA) cascade pathway. In particular, DHA-enriched beverages reduce the initial inflammatory response in neutrophil cells; such an effect is probably due to DHA that competes with amino acids as a substrate for cyclooxygenase 2 (COX-2) and 5- lipoxygenase (5-LOX), thereby reducing the production of inflammatory lipid mediators derived from AA [119]. Furthermore, the prostaglandins (PGE1 and PGE2) deriving from COX-1 and COX-2 cascade and are involved in the initiation and resolution of the inflammation process to be modulated by DHA-enriched beverages supplementation [116,120].

To date, only few studies report the effects of lipid-enriched beverage supplementation in athletes, most of them being carried out by Pons and colleagues. These authors have evaluated the supplementation of DHA-enriched drinks (1.14 g/d) on several physiological and biochemical markers in blood, erythrocytes, and peripheral mononuclear cells (PMBCs) of professional soccer players, after training and acute exercise [101,121]. The athletes were divided into an experimental group (EG), allowed to consume an isotonic CHO-electrolyte drink containing also 3% almond oil, 0.6% olive oil, and 0.2% DHA-S, and into a control group (CG), allowed to consume an isotonic CHO-electrolyte drink also containing 3% almond oil and 0.8% olive oil. DHA-S is an algal nutritional oil derived from *Schizochytrium* sp., containing a minimum of 35% of DHA and vitamin E as an antioxidant. The supplementation was carried out for 5 d/week, for 8 week. Blood samples were collected at the beginning, in resting conditions, and after 8 weeks of nutritional intervention during the training period, before and after a 2-h habitual training session. In the blood, they observed an increase in the plasmatic availability of DHA in non-esterified fatty acids (NEFAs) and tryglicerides (TGAs) and an increase in the PUFAs of NEFAs in the EG. Conversely, DHA-enriched drinks did not induce positive effects on the plasma biomarkers related to the oxidative balance, i.e., catalase and superoxide dismutase (SOD) enzymatic activities, and on malonyldialdehyde, a hallmark of lipidic peroxidation [101]. The effect of DHA-beverage supplementation was found to be tightly related to an increase in the plasma concentration of prostaglandin PGE2, thus demonstrating to have systemic anti-inflammatory effects [101]. When the focus was shifted to the erythrocytes, the authors found that DHA-enriched beverages enhanced the catalytic activity of SOD, with consequent reduction of the oxidative damage induced by training or exercise [121]. In addition, the DHA-enriched drinks significantly reduced serum levels of TNF-α and IL-6, pro-inflammatory cytokines produced in excess by PMBCs after an acute exercise, through the activation of the lipopolysaccharide (LPS) [119]. Finally, these functional beverages cooperated, along with training, to increase plasmatic PGE2 concentration with systemic anti-inflammatory effects [117].

Pons and colleagues evaluated also whether the DHA-enriched beverage supplementation could modulate oxidative stress and improve physical performance both in young (taekwondo) and senior male competitive athletes [99,110]. Both young and senior athletes were divided into a EG and into a CG, assuming the same DHA-based drinks quoted above, 5d/week for 5week, and blood sample collection followed the previous scheme. Moreover, the athletes performed maximal tests (stress test performed up to exhaustion at 90% VO_2_max in a hot environment) under controlled conditions before and after DHA-enriched beverage somministration. In this context, DHA supplementation did not affect any athletic performance-related parameters. In particular, body and skin temperature, plasma lactate concentration, time spent until exhaustion, and subjective fatigue perception (Borg index), considered markers of physical performance, were measured after the stress exercise test. Even the heat storage, reflecting the balance between metabolic heat production, heat absorbed from the environment, and the total body heat loss, was evaluated. All these physical performance markers results increased, in an overlapping manner, both in the control and in the experimental group, both in the young and in the elderly, except for the heat storage which instead was reduced in all groups. Therefore, DHA supplementation did not affect any of the parameters reported above, where even the duration of the exercise test (until exhaustion) results were not modified by this dietary supplementation in all groups. Only the subjective perception of effort, which can be modulated by other behavioral factors related to heat adaptation, was significantly reduced in senior compared to younger athletes, according to Reference [122]. These data do not differ from current literature which, to date, does not provide sufficient evidence to support a beneficial role of this dietary supplementation on physical performance. However, DHA supplementation positively influenced the composition of fatty acids, both at the plasma level and in erythrocytes, and in younger than in senior athletes. It also protected against oxidative damage in both groups. Finally, DHA-based beverages induced the expression of genes encoding for antioxidant enzymes in PMBCs after acute exercise in young athletes [99,110].

The DHA-enriched beverage supplementation also showed positive effects in the management and/or in the prevention of chronic inflammation associated with aging and non-communicable diseases [110]. In fact, Capò et al. recently demonstrated that these functional beverages were able to counteract the production of pro-inflammatory cytokines (Il-6 and TNF-α) and some adhesion molecules involved in the inflammatory process, such as molecule 3 of soluble intercellular adhesion (sICAM3) and soluble L-selectin (sL-selectin). All these species were produced in excess during exercise especially in younger compared to senior athletes [110].

Collectively, these studies suggest that DHA-enriched beverage supplementation might improve physical performance not in a direct fashion, i.e., not modifying parameters that are closely related to athletic performance, but indirectly by favoring post-exercise recovery, reducing cellular oxidative damage, and counteracting the production of pro-inflammatory molecules associated also with organ damage.

## 5. Functional Beverages Containing Amino Acids and Proteins

Athletes, as well as physically active subjects, need to assure adequate amounts of proteins to balance muscle protein synthesis (MPS) and breakdown (MPB). A daily protein intake ranging from 1.2–1.7 g/kg, corresponding to 10–12% of total energy, should ensure a positive nitrogen balance [40].

While protein supplementation has no effects on MPS during exercise [123,124], it is widely accepted that protein supply is highly effective on post-exercise MPS, particularly after endurance and/or RT [40]. In fact, according to the post-exercise anabolic window theory, the time window of between 30 min to 2 h after exercise is considered strategic not only for refueling muscle glycogen but also for boosting MPS [125,126]. Both processes seem to be linked by the putative insulin release via protein supply [126,127], but not all studies support this finding [40,120,121,122,123,124,125,126,127,128,129,130,131].

High intensity resistance training or interval-based activities induce several changes, at physiological and biochemical level, involving inflammatory markers (such as pro- and anti-inflammatory adipokines or high-sensitivity C-reactive protein), creatine kinase, myoglobin, oxidative stress, immune response, and muscle morphology [132,133,134,135,136,137,138]. These changes can affect the exercise-related physical demands, and hence post-workout nutrition has always been a main focus of researchers’ investigations.

Historically, early studies on protein supplementation took into account individual amino acids (AAs) or their mixtures [139,140], whereas more recent investigations focus on intact high-quality proteins [131,141,142,143,144,145,146,147]. It is known that a small amount (6 g) of essential AAs (EAAs; histidine, isoleucine, leucine, lysine, methionine, phenylalanine, threonine, tryptophan, and valine) are sufficient to stimulate MPS in a dose-dependent manner [148,149]. In particular, for a long time branched-chained AAs (BCAAs; leucine, isoleucine and valine) alone have been considered able to produce an anabolic response in skeletal muscles [150,151], but recently their ability has been questioned [152,153] suggesting a negative effect on muscle performance due to enhanced levels of blood ammonia following a BCAAs supplementation [152]. On the other hand, nonessential AAs (NEAAs) alone apparently are not necessary, even though a balanced mixture of EAAs and NEAAs is more effective than EAAs alone [140].

The composition of the EAAs mixture has also been analyzed and originally set as mirroring the composition of muscle proteins [148]. Successively, it was observed that each EAA could have a specific clearance rate, so that the corresponding uptake rate could not match the composition of the ingested mixture [149]. As for the pattern of ingestion, the initial speedup of MPS, determined by the EAA supply, is followed 2 h after by a return to the basal rate, even if the supplementation keeps a sustained high AA plasmatic concentration [148,154]. According to Borsheim and colleagues, the time of AA ingestion does not affect the effectiveness of the stimulus in young men after resistance exercise [149]; however, other reports indicate that the earlier the better, following the post-exercise anabolic window theory. In particular, the supplementation provided at the end of the exercise session worked better than 2 h after, in the resistance-trained elderly [155] and in aerobic-trained adult men [156].

Regarding AA availability from protein sources, the protein type and its digestion rate and kinetics have to be taken into account [157,158,159]. High-quality protein sources are milk, eggs, soy, wheat, and peas. Skimmed milk is considered the best natural protein beverage containing all EAAs required by humans and favoring MPS after resistance exercise [158,160,161,162]. There are two major categories of milk proteins: Caseins (80%), that coagulate at pH 4.6, and whey proteins (20%), that are soluble at pH 4.6. Although caseins are characterized by a slow digestion rate and speed of AA absorption (slow proteins) [146,163], they seem to inhibit MPB by 30% [164,165]. Such an effect, along with their high content in glutamine, make them quite popular among bodybuilders. In fact, caseins promote muscle build and are less prone to be used as an energy substrate. On the contrary, whey proteins are quickly digested and absorbed (fast proteins), and contain about 50% EAAs, half of which are BCAAs [166]. Compared to caseins, whey proteins are characterized by a higher biological value and boost more MPS [159,167]. In fact, 0.3 g/kg lean body mass of this soluble fraction is able to stimulate MPS up to 6 h following resistance exercise [168]. Additionally, whey proteins hydrolysate, constituted by di- and tri-peptides, are not only quickly digested, but also perform as gluconeogenic substrates contributing to the resynthesis of glycogen [144]. Both slow and fast milk proteins contribute similarly to the long-term adaptations of muscles to RT [146]. In fact, Fabre and colleagues showed that protein-containing beverages, having different fast-to-slow protein ratios, determined a similar effect on muscle strength (1-RM test) after 9 weeks of RT [146]. Finally, it is worth noting that whey proteins are beneficial for gut health and immune function. In particular, the lactoferrin and lactoferricin derived from milk have anti-microbial activity; furthermore, lysosome, lactoperoxidase, and diverse globulins and peptides may provide a synergistic protective activity against viral and bacterial infections mainly when chronic exercise partially suppresses the immune function [10].

Nowadays the market is crowded by a wide range of protein/AA powders to be diluted, as well as ready-to-drink protein beverages, often blended with other macronutrients (mainly CHO), micronutrients (vitamins, minerals), and/or flavors and sweeteners to get a more palatable final product. Moreover, some of them are 100% compatible with specific diet regimens, for example, functional beverages containing egg proteins, specifically egg albumin, are chosen for their high content of EAAs and for being lactose-free, whereas sport drinks containing soy, wheat, or pea proteins are the best vegetarian choice, being lactose- and cholesterol-free, the latter also being gluten-free.

Currently, among the different shelf-available products, mixed CHO- and protein-enriched functional (CHO-P) beverages are the most popular drinks due to their absorptive properties. Based on that, the most recent investigations are considering different protein/AA supplements in combination with CHO as the best nutrition aid in sport. The comparison among them is far from straightforward because of the different pattern of ingestion, CHO dosage, caloric content, and types of proteins are considered. McLellan and collegues reported that the ergogenic efficacy of CHO-P drinks, evaluated both during an acute bout of exercise and after an exercise to assess a subsequent endurance performance, relies on CHO supplementation rates [169]. In fact, when CHO delivery was sub-optimal (<60 g/h), then protein addition provided an ergogenic advantage to time-to-exhaustion and to time-trial performance tests. On the contrary, when CHO supplementation was at least 60 g/h, protein addition did not yield any ergogenic benefit [169]. Accordingly, Millard-Stafford and collegues found that additional calories (CHO or proteins) to CHO supplementation (at optimal rates) did not improve the performance after recovery (after 2 h or the following day) in runners, although the CHO-P drink attenuated muscle soreness compared to an isocaloric CHO beverage [131]. By testing a mixed supplementation during exercise, Highton and colleagues demonstrated that CHO-P beverages provide a small (2–3%) but significant advantage, in terms of covered distance and average running speed, in the latter stages of a multiple-sprint running performance [143]. These authors reported a mean ingestion rate of (52.7 ± 8.3 g/h CHO plus 17.6 ± 2.8 g/h protein) for CHO-P and of 70.2 ± 11.1 g/h for CHO; hence, the extra energy from protein might have been magnified by the sub-optimal delivery of CHO in the mixed drink. In addition, Breen and colleagues showed that CHO-P supplementation after exercise at a sub-optimal rate increased myofibrillar protein synthesis in cyclists more than CHO alone [142]. Such a result could have implications not only in the structural integrity of the contractile organ, but also in muscle hypertrophy if the post-endurance exercise protein ingestion is frequent.

Altogether, the current data suggest that further studies are needed to better clarify the mechanisms and to optimize the nutritional strategies through which CHO-P beverages are effective on performance and recovery.

## 6. Conclusions

Commercially available functional beverages addressing athletes and physically active subjects are formulated to answer to several purposes, including energy supply, electrolyte replacement, prevention of dehydration, pre-exercise and post-exercise hydration, in order to improve sports performance and minimize fatigue. Hence, popular sports drinks represent a compromise designed to meet the needs of most people in most different situations, and no single formulation will always be able to fulfill them all because of individual variability.

It is widely accepted that CHO-electrolyte functional sport drinks, particularly those containing Glc-Fru and sodium, can improve athletic performance by sustaining metabolism and optimizing water absorption. Evidence to integrate other components to improve sports performance is not clear yet; for example, the use of DHA-enriched drinks does not directly improve performance, but through the reduction of the exercise-induced oxidative damage, should favor post-exercise recovery. With regard to protein supplementation, protein sport drinks are mainly addressed to post-exercise recovery, but many aspects still need to be unraveled, above all if a co-ingestion with CHO is taken into account.

At last, it is important to remember that the global market of functional beverages is estimated in $US billions and is constantly growing. In fact, intense marketing efforts are continually made to encourage consumption, even when it is not needed. Many functional products have clean safety histories, but sometimes labels might not contain the right amount of the listed items, or miss some extra-ingredients, or be accidentally contaminated with allergens, and more concerns arise when youngers or intolerant subjects are targeted. Additional research in this field will help athletes and physically active subjects to safely choose the functional sport drink that better meets their needs.
